# Diagnostic Role of Carotid Intima-Media Thickness for Coronary Artery Disease: A Meta-Analysis

**DOI:** 10.1155/2020/9879463

**Published:** 2020-02-25

**Authors:** Dianmei Liu, Caiju Du, Weiguang Shao, Guifeng Ma

**Affiliations:** Imaging Center, Affiliated Hospital of Weifang Medical University, 2428 Yuhe Road, Kuiwen District, Weifang, 261031 Shandong Province, China

## Abstract

**Background:**

The present meta-analysis was conducted to confirm whether carotid intima-media thickness (IMT) could serve as an accurate diagnostic method for coronary artery disease (CAD).

**Methods:**

Databases of PubMed, Google Scholar, and Embase were searched for potential articles. The articles were selected according to inclusion criteria. Pooled sensitivity and specificity with corresponding 95% confidence interval (CI) were used to confirm the diagnostic role of IMT for CAD. *I*^2^ and *P* value were used to assess the existence of heterogeneity. *I*^2^ and *P* value were used to assess the existence of heterogeneity.

**Results:**

22 eligible articles were selected in the present meta-analysis. Pooled sensitivity and specificity of IMT for diagnosing CAD were 0.68 (0.57–0.77) and 0.70 (0.64–0.75), respectively. The corresponding AUC was 0.74 (0.70–0.78). Subgroup analyses based on cutoff value of IMT were performed. A cutoff value of 1 mm was demonstrated to be much more accurate diagnostic criteria for CAD (sensitivity: 0.66; specificity: 0.79; AUC: 0.80). Sensitivity analysis indicated that the pooled results were robust. Deek's funnel plot indicated no significant publication bias (*P* value were used to assess the existence of heterogeneity.

**Conclusion:**

Carotid IMT may serve as an accurate diagnostic tool. A cutoff value of 1 mm seems to provide much more accurate diagnostic results for CAD.

## 1. Introduction

Coronary artery disease (CAD) is regarded as one cause of death around the world. Hypertension, diabetes, fibrinogen, low-density lipoprotein cholesterol, and smoking are common risk factors for CAD [1]. These traditional risk factors are reported to be weak predictors of CAD [2]. Coronary angiography is commonly performed before valve surgery in most patients older than 35 years old [3]. It remains the gold standard for assessing the degree of coronary atherosclerosis. However, this invasive method is related with nonnegligible morbidity, especially for the patients with inconstant hemodynamic variables. Thus, it is urgent to explore noninvasive screening method for diagnosing CAD patients.

More recently, intima-media thickness (IMT) of the common carotid artery has been suggested as quick, noninvasive, and reproducible marker for CAD [4–7]. IMT is usually assessed by quantifying the distance between the echogenic media-adventitia layer and the echogenic lumen-intima layer with B-mode ultrasound images [8]. It is regarded as a marker predicting early stages of atherosclerotic process and related with the occurrences of cardio-/cerebrovascular events and cardiovascular outcomes [9–11]. In addition, it has been demonstrated to be correlated with the coronary risk factors including smoking, sex, diabetes, hypertension, and cholesterol [12–14]. Besides, IMT could predict the severity of CAD. A previous study by Kablak-Ziembicka et al. concluded that CAD patients had high IMT compared with healthy controls. Moreover, patients with more advanced CAD had higher IMT [15]. Although a significant association between IMT and the development of CAD had been observed in several studies, the exact role of IMT in the clinical screening of CAD has yet to be established.

Our meta-analysis was performed to extract a definitive conclusion about the diagnostic role of IMT for CAD. The analysis contributed to extensive application of IMT in a clinic.

## 2. Materials and Methods

### 2.1. Article Search

The potential articles were searched on the databases of PubMed, Google Scholar, and Embase up to April 2017. The keywords were “carotid intimamedia” OR “intima-media thickness” AND “coronary artery disease” OR “CAD” OR “myocardial infarction” OR “ischaemic heart disease.” Their combinations of these keywords were also used during search. The references of obtained articles were checked for potential valuable articles. Only the articles in English were considered.

### 2.2. Inclusion Criteria

We selected the articles according to inclusion criteria. The inclusion criteria were as follows: (a) carotid intima-media thickness (IMT) was used to diagnose CAD, myocardial infarction, or ischaemic heart disease and (b) numbers of patients and controls, sensitivity, specificity or true positive (TP), false negative (FN), false positive (FP), and true negative (TN) were provided. The review articles and case reports were excluded from the analysis.

### 2.3. Data Extraction

The following data was extracted: name of first author; year of publication; country; number of patients and healthy controls; cutoff value; area under the curve (AUC) of IMT; diagnostic TP, FN, FP, and TN; or any data available for calculating them. Two authors were responsible for extracting data, and they independently performed the process. For the articles with the same population, the much more comprehensive one was selected.

### 2.4. Statistical Analysis

Data analysis was performed with Stata 12.0 software. Quality Assessment of Diagnostic Accuracy Studies (QUADAS-2) criteria were applied to evaluate the quality of the included articles. Diagnostic sensitivity and specificity with corresponding 95% confidence interval (CI) were applied to figure out the diagnostic role of IMT for CAD. *I*^2^ and *P* value were used to assess the existence of heterogeneity. *I*^2^ ≥ 50% or *P* < 0.05 indicated significant heterogeneity. The AUC of summary receiver operating characteristics (SROC) was used to evaluate the diagnostic accuracy of IMT for CAD. Sensitivity analysis was performed to evaluate the robustness of pooled outcomes. Deek's funnel plot was adopted to assess potential publication bias.

## 3. Results

### 3.1. Article Selection Process

**Figure 1:**
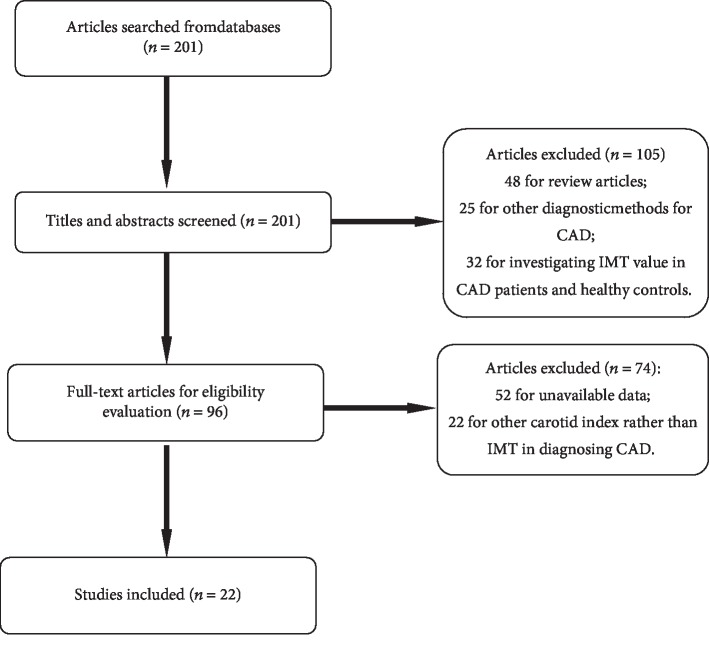
Selection process of eligible articles. A total of 22 articles were selected in the present meta-analysis.

**Figure 2:**
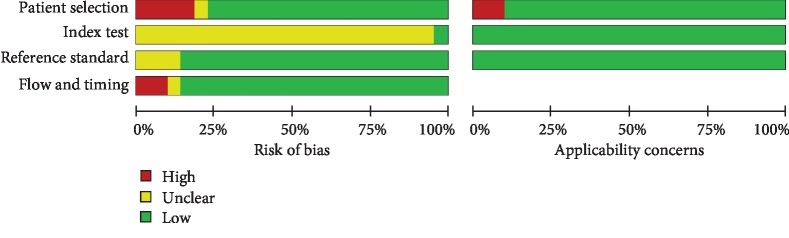
QUADAS criteria for quality evaluation of included studies.

A total of 201 relevant articles were obtained after search. Then, the abstract and titles were screened, and 105 articles were removed for review articles, other diagnostic methods for CAD, and investigating IMT value in CAD patients and healthy controls. For the remaining articles, their full texts were evaluated for eligibility. During evaluation, 74 articles were removed for unavailable data and other carotid index rather than IMT in diagnosing CAD. Finally, 22 eligible articles were selected [7, 16–36] (Figure 1). The basic information of selected articles is shown in Table 1. The quality of included studies is shown in Figure 2.

### 3.2. Diagnostic Accuracy of IMT for CAD

**Figure 3:**
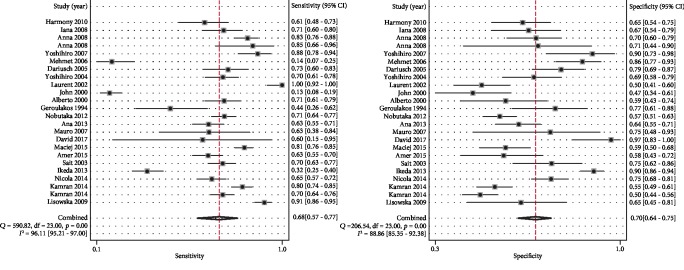
Diagnostic sensitivity and specificity of carotid IMT for CAD. The sensitivity and specificity were 0.68 and 0.70, respectively.

**Figure 4:**
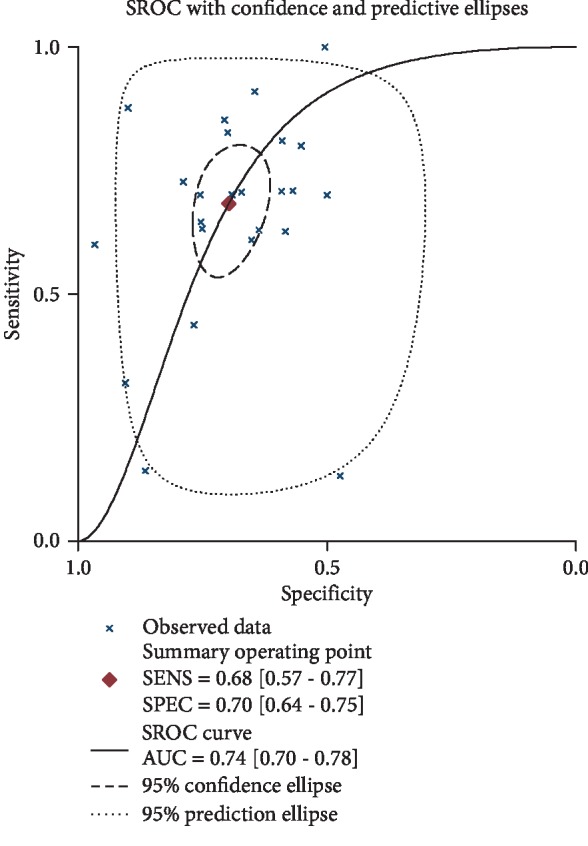
SROC analysis. AUC was 0.74 (95% CI: 0.70–0.78).

As shown in Figure 3, the sensitivity and specificity of IMT for diagnosing CAD were 0.68 (0.57–0.77) and 0.70 (0.64–0.75), respectively. The corresponding AUC was 0.74 (0.70–0.78) (Figure 4).

### 3.3. Subgroup Analyses Based on the Cutoff Value of IMT

The results are shown in Table 2. If the cutoff value of IMT ≤ 0.8 mm, the diagnostic sensitivity and specificity of IMT for diagnosing CAD were 0.66 (0.15–0.95) and 0.66 (0.51–0.78). The AUC was 0.69 (0.64–0.73). If the cutoff value of IMT is between 0.8 and 1 mm, the diagnostic sensitivity and specificity of IMT for diagnosing CAD were 0.71 (0.64–0.77) and 0.67 (0.58–0.75). The corresponding AUC was 0.75 (0.71–0.78). If the cutoff value of IMT > 1 mm, the diagnostic sensitivity and specificity of IMT for diagnosing CAD were 0.66 (0.48–0.80) and 0.79 (0.69–0.86). The AUC was 0.80 (0.76–0.83).

### 3.4. Sensitivity Analysis

Sensitivity analysis was performed, and the analysis indicated that the pooled results were robust.

### 3.5. Publication Bias Detection

**Figure 5:**
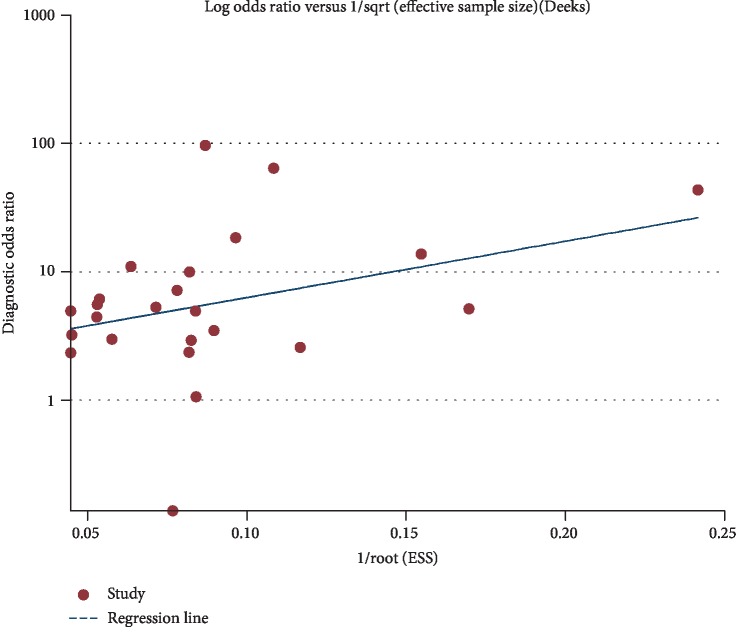
Deek's funnel plot for publication bias. No significant publication bias was observed in the present meta-analysis (*P* = 0.195).

Deek's funnel plot was drawn to detect potential publication bias. No significant publication bias was observed (*P* = 0.195) (Figure 5).

## 4. Discussion

It is commonly thought that atherosclerosis is a generalized disease, which mainly occurs in the early decades of life [37]. Coronary and carotid arteries are the two most common sites related with atherosclerosis [38]. The relationship of coronary and carotid atherosclerosis has been confirmed [39]. Carotid IMT is regarded as a marker of atherosclerosis. Previous studies have suggested that IMT would increase with hypertension, diabetes mellitus, hyperlipidaemia, age, sex, percentile, and population and other factors that are closely related with CAD [9, 40–42].

There are many methods to evaluate the arteries' condition. Coronarography is the golden standard for diagnosis of coronary artery atherosclerosis. However, coronarography is invasive with a definite risk. IMT is a well-described marker for cardiovascular disease, and enhanced IMT is correlated with the development of CAD and stroke [43, 44]. IMT more than 1 mm is correlated with a twofold increased risk of CAD in men and fivefold increased risk in women [1].

Until now, the diagnostic role of IMT for CAD is controversial. In the study by Lisowska et al., the relationship of IMT and the extent of CAD was evaluated, and the results indicated that the diagnostic sensitivity of IMT was 91%, while the specificity was 65% [16]. Belhassen et al. assessed the value of carotid IMT in ruling out significant CAD in patients for heart valve surgery and concluded that IMT had 100% sensitivity and 50% specificity in diagnosis of CAD [29]. Kanadaşi et al. obtained similar outcome [32]. In their analysis, the diagnostic sensitivity was merely 14.3%, while specificity was 86.4%. However, there exists remarkable difference in the results about sensitivity and specificity. The research conducted by Zhang et al. reported that the cutoff value of IMT was 1 mm and the corresponding sensitivity and specificity were 31.91% and 90.52% [4]. Murphy et al. found that the sensitivity and specificity of carotid IMT ≥ 0.9 mm were 50% and 96% [23]. In contrast, the results of Matsushima et al. were moderate [30]. A cutoff value of 0.88 mm of IMT provided 88% sensitivity and 90% specificity. The results of our analysis suggested that the diagnostic sensitivity and specificity were 68% and 70%. The corresponding AUC was 0.74. Meanwhile, subgroup analysis by the cutoff value of IMT was also performed. A cutoff value of IMT ≤ 0.8 mm provided 66% sensitivity and 66% specificity. A cutoff value of IMT between 0.8 and 1 mm provided 71% sensitivity and 67% specificity. A cutoff value of IMT > 1 mm provided 66% sensitivity and 79% specificity. Our analysis was performed based on 22 relevant articles. The involved countries included Poland, Iran, Italy, Japan, Turkey, Canada, Ireland, Portugal, UK, Greece, Germany, and America. Moreover, these studies were all well-designed researches. The processes of extracting data and analyzing data were all carefully performed by two independent authors. Thus, the pooled results were credible and reliable. However, the limitations should be mentioned. First, there were less studies involving Asian population compared with Caucasian population, which might affect the applicability of the results in Asian population. Second, there existed significant heterogeneity in the sensitivity and specificity analyses. It might result from the difference in the territory, performance, patients' status, and cutoff value of IMT.

## 5. Conclusion

In conclusion, carotid IMT is suggested to be a practical tool for screening CAD. The results will help us understand the clinical status of IMT in diagnosis of CAD.

## Figures and Tables

**Table 1:** Basic information of included articles.

Author	Year	Country	Sample size	Cutoff (mm)	AUC
Lisowska	2009	Poland	231	—	—
Kamran	2014	Iran	500	Right: 0.82; left: 0.85	Right: 0.70; left: 0.70
Nicola	2014	Italy	446	0.80	—
Ikeda	2013	Japan	370	1	0.648
Sait	2003	Turkey	233	—	—
Amer	2015	Canada	217	0.82	0.611
Maciej	2015	Poland	412	0.76	0.716
David	2017	Ireland	35	0.9	
Mauro	2007	Italy	35	1	0.885
Ana	2013	Portugal	300	0.85	0.638
Nobutaka	2012	Japan	501	0.9	0.791
Geroulakos	1994	UK	75	0.85	—
Alberto	2000	Italy	150	0.83	—
John	2000	Greece	224	0.8	—
Laurent	2002	France	152	0.55	—
Yoshihiro	2004	Japan	205	1.1	—
Dariusch	2005	Germany	151	1	—
Mehmet	2006	Turkey	144	0.8	—
Yoshihiro	2007	Japan	103	0.88	0.92
Anna	2008	Poland	277	0.933; 1.075	0.817
Iana	2008	Bulgaria	146	0.81	0.71
Harmony	2010	America	150	0.9	—

Note: UK: United Kingdom; IMT: intima-media thickness; CAD: coronary artery disease; AUC: area under the curve.

**Table 2:** Subgroup analyses.

Subgroup	Sensitivity	95% CI	Specificity	95% CI	AUC	95% CI
≤0.8 mm	0.66	0.15–0.95	0.66	0.51–0.78	0.69	0.64–0.73
0.8–1 mm	0.71	0.64–0.77	0.67	0.58–0.75	0.75	0.71–0.78
≥1 mm	0.66	0.48–0.80	0.79	0.69–0.86	0.80	0.76–0.83

## References

[1] Chambless L. E., Heiss G., Folsom A. R. (1997). Association of coronary heart disease incidence with carotid arterial wall thickness and major risk factors: the Atherosclerosis Risk in Communities (ARIC) Study, 1987-1993. *American Journal of Epidemiology*.

[2] Berdah J., Luxereau P., Vahanian A. (1988). Predictive factors of coronary lesions in aortic stenosis in adults. *Archives des Maladies du Coeur et des Vaisseaux*.

[3] Bonow R. O., Carabello B., de Leon A. C. (1998). ACC/AHA guidelines for the management of patients with valvular heart disease. Executive summary. A report of the American College of Cardiology/American Heart Association Task Force on Practice Guidelines (committee on management of patients with valvular heart disease). *The Journal of Heart Valve Disease*.

[4] Zhang Y., Guallar E., Qiao Y., Wasserman B. A. (2014). Is carotid intima-media thickness as predictive as other noninvasive techniques for the detection of coronary artery disease?. *Arteriosclerosis, Thrombosis, and Vascular Biology*.

[5] Balta S., Aparci M., Ozturk C., Unlu M., Celik T. (2015). Carotid intima media thickness can predict coronary artery disease. *International Journal of Cardiology*.

[6] Chang C. C., Chang M. L., Huang C. H., Chou P. C., Ong E. T., Chin C. H. (2013). Carotid intima-media thickness and plaque occurrence in predicting stable angiographic coronary artery disease. *Clinical Interventions in Aging*.

[7] Timóteo A. T., Carmo M. M., Ferreira R. C. (2013). Carotid intima-media thickness and carotid plaques improves prediction of obstructive angiographic coronary artery disease in women. *Angiology*.

[8] Burke G. L., Evans G. W., Riley W. A. (1995). Arterial wall thickness is associated with prevalent cardiovascular disease in middle-aged adults. The Atherosclerosis Risk in Communities (ARIC) Study. *Stroke*.

[9] Davis P. H., Dawson J. D., Riley W. A., Lauer R. M. (2001). Carotid intimal-medial thickness is related to cardiovascular risk factors measured from childhood through middle age: the Muscatine study. *Circulation*.

[10] O'Leary D. H., Polak J. F., Kronmal R. A., Manolio T. A., Burke G. L., Wolfson SK Jr (1999). Carotid-artery intima and media thickness as a risk factor for myocardial infarction and stroke in older adults. Cardiovascular Health Study Collaborative Research Group. *The New England Journal of Medicine*.

[11] Silvestrini M., Cagnetti C., Pasqualetti P. (2010). Carotid wall thickness and stroke risk in patients with asymptomatic internal carotid stenosis. *Atherosclerosis*.

[12] Kirhmajer M. V., Banfic L., Vojkovic M., Strozzi M., Bulum J., Miovski Z. (2011). Correlation of femoral intima-media thickness and the severity of coronary artery disease. *Angiology*.

[13] Grau M., Subirana I., Agis D. (2012). Grosor intima-media carotideo en poblacion española: valores de referencia y asociacion con los factores de riesgo cardiovascular. *Revista Española de Cardiología*.

[14] Evensen K., Sarvari S. I., Rønning O. M., Edvardsen T., Russell D. (2014). Carotid artery intima-media thickness is closely related to impaired left ventricular function in patients with coronary artery disease: a single-centre, blinded, non-randomized study. *Cardiovascular Ultrasound*.

[15] Kablak-Ziembicka A., Tracz W., Przewlocki T., Pieniazek P., Sokolowski A., Konieczynska M. (2004). Association of increased carotid intima-media thickness with the extent of coronary artery disease. *Heart*.

[16] Lisowska A., Musiał W. J., Lisowski P., Knapp M., Małyszko J., Dobrzycki S. (2009). Intima-media thickness is a useful marker of the extent of coronary artery disease in patients with impaired renal function. *Atherosclerosis*.

[17] Azarkish K., Mahmoudi K., Mohammadifar M., Ghajarzadeh M. (2014). Mean right and left carotid intima-media thickness measures in cases with/without coronary artery disease. *Acta Medica Iranica*.

[18] Gaibazzi N., Rigo F., Facchetti R. (2014). Ultrasound carotid intima–media thickness, carotid plaque and cardiac calcium incrementally add to the Framingham Risk Score for the prediction of angiographic coronary artery disease: a multicenter prospective study. *International Journal of Cardiology*.

[19] Ikeda N., Saba L., Molinari F. (2013). Automated carotid intima-media thickness and its link for prediction of SYNTAX score in Japanese coronary artery disease patients. *International Angiology*.

[20] Alan S., Ulgen M. S., Ozturk O., Alan B., Ozdemir L., Toprak N. (2003). Relation between coronary artery disease, risk factors and intima-media thickness of carotid artery, arterial distensibility, and stiffness index. *Angiology*.

[21] Johri A. M., Behl P., Hétu M. F. (2016). Carotid ultrasound maximum plaque height–a sensitive imaging biomarker for the assessment of significant coronary artery disease. *Echocardiography*.

[22] Haberka M., Gąsior Z. (2015). A carotid extra-media thickness, PATIMA combined index and coronary artery disease: comparison with well-established indexes of carotid artery and fat depots. *Atherosclerosis*.

[23] Murphy D. J., Crinion S. J., Redmond C. E. (2017). Diagnostic accuracy of carotid intima media thickness in predicting coronary plaque burden on coronary computed tomography angiography in patients with obstructive sleep apnoea. *Journal of Cardiovascular Computed Tomography*.

[24] Amato M., Montorsi P., Ravani A. (2007). Carotid intima-media thickness by B-mode ultrasound as surrogate of coronary atherosclerosis: correlation with quantitative coronary angiography and coronary intravascular ultrasound findings. *European Heart Journal*.

[25] Ikeda N., Kogame N., Iijima R., Nakamura M., Sugi K. (2012). Carotid artery intima-media thickness and plaque score can predict the SYNTAX score. *European Heart Journal*.

[26] Geroulakos G., O’Gorman D. J., Kalodiki E., Sheridan D. J., Nicolaides A. N. (1994). The carotid intima-media thickness as a marker of the presence of severe symptomatic coronary artery disease. *European Heart Journal*.

[27] Balbarini A., Buttitta F., Limbruno U. (2000). Usefulness of carotid intima-media thickness measurement and peripheral B-mode ultrasound scan in the clinical screening of patients with coronary artery disease. *Angiology*.

[28] Lekakis J. P., Papamichael C. M., Cimponeriu A. T. (2000). Atherosclerotic changes of extracoronary arteries are associated with the extent of coronary atherosclerosis. *The American Journal of Cardiology*.

[29] Belhassen L., Carville C., Pelle G. (2002). Evaluation of carotid artery and aortic intima media thickness measurements for exclusion of significant coronary atherosclerosis in patients scheduled for heart valve surgery. *Journal of the American College of Cardiology*.

[30] Matsushima Y., Kawano H., Koide Y. (2004). Relationship of carotid intima-media thickness, pulse wave velocity, and ankle brachial index to the severity of coronary artery atherosclerosis. *Clinical Cardiology*.

[31] Haghi D., Papavassiliu T., Hach C. (2005). Utility of combined parameters of common carotid intima–media thickness or albuminuria in diagnosis of coronary artery disease in women. *International Journal of Cardiology*.

[32] Kanadaşi M., Cayli M., San M. (2006). The presence of a calcific plaque in the common carotid artery as a predictor of coronary atherosclerosis. *Angiology*.

[33] Matsushima Y., Takase B., Uehata A. (2007). Comparative predictive and diagnostic value of flow-mediated vasodilation in the brachial artery and intima media thickness of the carotid artery for assessment of coronary artery disease severity. *International Journal of Cardiology*.

[34] Kablak-Ziembicka A., Przewlocki T., Tracz W. (2008). Carotid intima-media thickness in pre- and postmenopausal women with suspected coronary artery disease. *Heart and Vessels*.

[35] Simova I., Denchev S. (2008). Endothelial functional and structural impairment in patients with different degrees of coronary artery disease development. *Heart and Vessels*.

[36] Reynolds H. R., Steckman D. A., Tunick P. A., Kronzon I., Lobach I., Rosenzweig B. P. (2010). Normal intima-media thickness on carotid ultrasound reliably excludes an ischemic cause of cardiomyopathy. *American Heart Journal*.

[37] Stary H. C. (1989). Evolution and progression of atherosclerotic lesions in coronary arteries of children and young adults. *Arteriosclerosis*.

[38] Hodis H. N., Mack W. J., LaBree L. (1998). The role of carotid arterial intima-media thickness in predicting clinical coronary events. *Annals of Internal Medicine*.

[39] Hulthe J., Wikstrand J., Emanuelsson H., Wiklund O., de Feyter P. J., Wendelhag I. (1997). Atherosclerotic changes in the carotid artery bulb as measured by B-mode ultrasound are associated with the extent of coronary atherosclerosis. *Stroke*.

[40] Sun Y., Lin C. H., Lu C. J., Yip P. K., Chen R. C. (2002). Carotid atherosclerosis, intima media thickness and risk factors: an analysis of 1781 asymptomatic subjects in Taiwan. *Atherosclerosis*.

[41] Ciccone M. M., Bilianou E., Balbarini A. (2013). Task force on: ‘early markers of atherosclerosis: influence of age and sex’. *Journal of Cardiovascular Medicine*.

[42] Ciccone M. M., Balbarini A., Teresa Porcelli M. (2011). Carotid artery intima-media thickness: normal and percentile values in the Italian population (camp study). *European Journal of Cardiovascular Prevention and Rehabilitation*.

[43] Simon A., Gariepy J., Chironi G., Megnien J. L., Levenson J. (2002). Intima-media thickness: a new tool for diagnosis and treatment of cardiovascular risk. *Journal of Hypertension*.

[44] Simon A., Megnien J. L., Chironi G. (2010). The value of carotid intima-media thickness for predicting cardiovascular risk. *Arteriosclerosis, Thrombosis, and Vascular Biology*.

